# Participation, community and affirmation: parent accounts of supporting transgender children in sport and dance participation

**DOI:** 10.3389/fspor.2026.1768522

**Published:** 2026-03-23

**Authors:** Cris Townley

**Affiliations:** Transforming Early Education and Child Health, Western Sydney University, Penrith, NSW, Australia

**Keywords:** adolescents and children, cisnormativity, dance, parent knowledge, participation and inclusion, sport, transgender (binary and non-binary)

## Abstract

**Introduction:**

Transgender (trans) young people participate in sport at lower rates than their cisgender peers. Whilst barriers and enablers to participation have been identified for trans adolescents and adults, we know little about the participation in sport of trans children. Parent accounts of supporting trans children are a rich source of knowledge about accessing health and education, but to date have had little to say about participation in physical activity.

**Methods:**

Data comprises interviews with 16 parents of trans children and young people in Australia about how they have supported their children to participate in physical activity such as sport and dance, and how they navigate these community environments.

**Results:**

We find that children communicate and affirm their gender through their choice of sport or dance, and what they wear when participating. Parents undertake a significant amount of work as allies for their children in community sport and dance environments. Limitations on participation are the increased importance of competition as children get older and the impact on children's mental health of their broader experience as trans people in the world. Access to inclusive sport and dance environments for trans children enables community participation and gender affirmation. Kindness in supportive community is important, with competition much less so.

**Discussion:**

Sport and dance continue to be dominated by cis-normative gender binary practices including uniforms and administrative processes. As a result, opportunities for non-binary participation are restricted. Deploying normative resistance and inventive pragmatism, parents navigate balancing privacy and disclosure to navigate access to participation and find their place in a community.

## 1 Introduction

Participation in physical activity by children and young people is beneficial for physical and mental health, cognitive functioning and academic achievement (1, 2). However, transgender (trans) young people have lower participation rates than their cisgendered peers (3). A number of barriers and facilitators to participation in sports have been identified for young trans adults (4) and adolescents (5). However, there is a dearth of literature examining the experiences of trans children under 12 (6). I am using the term “trans” to encompass those whose gender identity does not align with the sex and gender that they were assigned at birth, including both binary trans people (who identify as boys/men or girls/women) and non-binary people. I also use the term “transitude”, to refer to the state of being trans and implying a non-medicalised perspective (7).

At a societal level there is divisive debate, for example in the media, about trans adults participating in competitive sport (8), particularly focused on trans women (9). Law and public policy governing trans participation in sport is often restrictive and contested, for example in the United States, and impacts on college and high school sports (10). Since this wider cultural context of division and hostility to trans people in sport impacts on the participation of trans people in high school and higher education (10) it is likely to have an impact on trans children too.

In contrast, in Australia, guidelines and resources developed in partnership between Sports Australia, the Australian Human Rights Commission and eight major national sporting bodies, support the inclusion of trans children in sport (11). A conceptual separation of competitive sport from community-based sport, particularly for children under 12, allows discrimination against trans children to be made unlawful. Dance is an additional form of physical activity popular with children. Whilst there are examples of dance communities that are moving towards trans inclusivity for adults (12), there is a lack of research into the inclusion of trans children in dance.

Insight into the experiences of trans children and young people can be gained by listening to parent accounts. For most children, it is their parents who navigate access to institutions and activities with them. Although there is a body of work on parent advocacy and support of their trans children and young people (13–15), there is little to date in parent accounts that addresses or informs their children's participation in sport or dance. Furthermore, little is known about the participation of trans children under 12 in such physical activity, or children and young people outside of school settings (16).

### 1.1 Participation of trans children in physical activity

A recent scoping review (17) sought to identify existing literature relating to physical activity interventions, participation and experiences for trans children, aged 10 to 25. They found 12 papers, none of which reported on children below 13 years old, with one that included parents in the study participants. This suggests a lack of knowledge about the participation in physical activity for young trans children, and an opportunity to investigate parent knowledge of facilitating this participation. The scoping review identified a number of findings. Overall, trans youth participated in sport to a lesser degree than cisgender youth. Barriers to participation included inadequate change facilities, body dissatisfaction, fears about being identified as trans, and of not being accepted. Many trans youth reported poor experiences of participating in sport, and these contributed to a fear of transphobia which was a barrier to further participation. The authors also found that athletic clothing is a barrier to participation, particularly where youth are prevented from wearing clothing associated with their preferred gender. Whilst trans youth preferred individual sports, there were exceptions where the clothing was a barrier, for example clothing for swimming and running. Lastly, the review found evidence that sports team participation was associated with better mental health for trans adolescents (18), and that there is a correlation between poorer mental health and being excluded from sport. Dance was not mentioned in the review results as an activity for trans young people.

Participation rates for trans adolescents vary for different trans identities. A large study in Minnesota analysed data on sport and physical activity reported by almost 120,000 adolescents, including 1,756 trans (binary and non-binary) adolescents and 2,105 adolescents questioning their gender (3). The study found that whilst over half of cisgender youth participated in sport, only a third of youth questioning their gender and a quarter of trans youth did so. Participation rates also varied among trans youth. Those trans young people with highest participation rates were non-binary, assigned male at birth adolescents, where just under one third participated in team sports. One in five non-binary, assigned female at birth adolescents played team sports as did one in five young trans women and, with the lowest participation rates, one in six young trans men. Unfortunately, the study (3) did not report on cisgender youth participation by gender so we cannot compare trans young men with cisgender young men, nor trans young women with their cisgender counterparts. Neither is it clear as to whether dance is included in the definition of sport and physical activity about which young people were asked. It has been found that participation reduces after gender disclosure (19). Greenspan et al. (20) suggest that this may be because gender disclosure precedes medical transition, so the young person's body shape is not yet as desired.

There is evidence that trans young people seek opportunities for sport and physical activity. A nuanced view of participation in Australia is explored by Storr et al. (21) through interviews with 13 (binary) trans, non-binary and gender diverse young people in Australia, aged 17 to 24. Most wanted to participate in sport and physical activity, recognising the community connection and health benefits that this brings. Almost all participants spoke about previous experiences of discrimination in their participation in sport, and of sports environments as heteronormative, with hyper-masculine behaviour dominating. This culture had left many of the young people feeling that they did not have the ability to play sport. They sought greater visibility of sexuality and gender diverse sports leaders, inclusive policies, and training for coaches and sports teachers to reduce the self-exclusion of sexuality and gender diverse people in sport.

Parental involvement in children's sport is increasing (22). However, little is known as to how parents of trans children navigate barriers to and possibilities for participation, especially in out of school settings. This paper contributes to filling the gap in our knowledge of sports participation for trans children and young people, particularly those under 12. This paper takes a sociological approach to exploring and understanding parent accounts of navigating sports environments with and for their trans children and adolescents.

### 1.2 Theoretical framework

Sport and dance are structured around a cis-normative gender binary, where there are underlying assumptions that people can be categorised as either male or female, and these categorisations align with gender assigned at birth (12, 23). While trans young people want to participate in sport (21), the embedded cis-normative gender binary in sports environments results in the exclusion of many trans adolescents (15). Trans children and young people navigating cis-normative binary environments are both invisibilised, where trans identities are unspoken, and at the same time hypervisible, as people who can't easily be categorised into cisnormative structures, or ways of organising the world (24).

This in/visibility and the dichotomous structure of the public/private sphere where cis-normativity privileges cis identities means trans people have to negotiate whether and how to pass in the public sphere (25) in order to “be recognised as human” (26) and therefore to access social participation. Passing, that is, being understood by others to be gendered in the binary gender of choice rather than that assigned at birth, renders transness invisible and can make life easier. However, visibility and celebration are dangerous and hard work. Being trans is neither completely open, nor totally secret, but “the process of making oneself known (or intelligible) as a trans subject can be thought of as a reiterative practice” with “simultaneous processes of disclosure and concealment” (26).

Giddens' structuration theory provides a means of conceptualizing parent work as “the often delicate and subtle interlacings of reflexively organized action and institutional constraint” (27). People in social systems exercise agency by drawing on the rules and resources and practices that drive behaviour. Agency is constrained by these rules, but at the same time it is agency that can change the social rules of structure. This agency can change or reproduce the structure of a social system or institution. In order to explore how this interplay of agency and structure can be navigated with respect to trans identities, I use the conceptual tools of “normative resistance “ and “inventive pragmatism” (28). Normative resistance is the deployment of conscious strategies in making choices that are not socially sanctioned or celebrated, and inventive pragmatism are actions that manipulate existing social structures to access resources in the course of the navigation.

### 1.3 Research questions

This paper reports on what we can learn from parent accounts about supporting trans children and young people to participate in sport. Particularly, what strategies do parents deploy in navigating agency and structure to support the participation of their trans children in sport? Building on this, what are the possibilities for more inclusive sporting policies and practices for trans children and young people?

## 2 Materials and methods

### 2.1 Participants and procedure

This paper is a subset of a wider project where 18 parents of trans children and young people were interviewed about the journey of supporting their trans child in education, health, and other services. This paper reports on the data from 16 parents who discussed participation in sport and dance. The semi structured interviews explored parents' interactions with a range of support services and institutions including health and education (15). I conducted this project in partnership with two networks of parents of trans children, of which I am also a member. My positionality as non-binary, and the parent of a trans young person who participated in a range of sport and dance communities as a child and adolescent, gave me an initial broad understanding of the area and a level of trust with participants on which to build. Recruitment was undertaken through these networks, and then via snowball sampling. This approach meant that participating parents already had access to support, and were likely to be affirming of their children. Project design and recruitment was facilitated by my membership of these networks. Whilst a potential disadvantage is that my insider status made it difficult to see perspectives outside those of affirming parents, centring affirming parent accounts is at the same time a strength of this research.

This research sought participation from parents rather than children for two reasons. Firstly, through collective peer support, parents have built a large body of knowledge about how to support their trans children across all areas of their lives, which this research seeks to make visible. Secondly, trans children face a burden of explaining themselves to the world in their daily lives that I did not wish to add to through research participation, where there was an alternative. Parents were offered the opportunity to review their interview transcript, and there were no requests for amendments. An important ethical consideration was protecting the anonymity of parents and their children. This was enhanced by the pseudonyms in this paper being different to pseudonyms attributed to these interviews in previous publications in order to protect the anonymity of participants, through reducing the possibility of cross-referencing data about individual families. Human ethics approval was obtained from the Human Research Ethics Committee at Western Sydney University, approval number H15457.

### 2.2 Data

The interview data is from 16 parents across 13 interviews, covering the experiences of 13 children and young people. The mean duration of these interviews was 85 minutes. The children that parents spoke about were aged between 8 and 21 at time of interview, and between 2 and 16 at the time that they communicated their trans identity. Of the 13 children and young people, six were girls, four boys and three non-binary, and eight interviews discussed participation in sport or dance for children under the age of 12.

All quotes have been reported using parent pseudonyms, and all quotes are taken from parents' accounts. Quotes are also attributed by identifying the age and pronouns of the child. “Preschooler” refers to a person who has not started school, aged up to 5 years old. “Child” refers to a person aged 6 to 12. “Youth” refers to a person aged 13 to 18. Age is attributed as at the time of the anecdote that is the subject of the reflection in the data.

### 2.3 Analysis

The interviews were transcribed using transcription software (29), then manually checked. The transcripts were coded in NVIVO (30). Analysis of the wider data set demonstrated that conversations about sport and dance emerged as a significant topic for parents. Data coded to the broad content category of sport and dance was extracted from NVIVO. This sport and dance data was thematically coded using a Big Q approach (31). This was done by initial coding to the themes *parent ally work* (subthemes administrative burden, stealth or communicate, physical presence); *binary gendered sports* (change rooms and facilities, what to wear?, non-binary participation, gender affirmation in community); and *limits of participation* (competition and medical journey, mental health opt out and sport in queer contexts). Data was printed out on paper organised in these categories and read through numerous times over a period of several months and annotated to support deep thinking. A process of iterative sorting and writing of the analysis refined the codes to *communicating gender*, *navigating disclosure and privacy*, the *kindness of community*, and “*give it a red hot crack”* (competition). “A red hot crack” is an Australian term that means your best effort. A quantitative assessment was made of the percentage of data (by wordcount in NVIVO) that discussed participation of children categorised as either girl, boy or non-binary.

## 3 Results and discussion

Although just under half (46%) of the children whose experiences are explored in the dataset were girls, 77% of the text coded to sport and dance was from parents of (trans) girls, 15% from parents of (trans) boys and 8% from parents of non-binary children and young people. This may indicate that parental work to support participation of trans children is more intensive for parents of girls. The data demonstrates that dance is an important example of physical activity and social participation for families. Parents spoke about dance and sport in similar ways, and dance was a common preference for trans girls, as it is for cis girls (32). The results of the thematic analysis are presented through four themes. These are the ways that sports participation can be used to communicate gender, navigating disclosure and privacy, the kindness of community, and competition.

### 3.1 Communicating gender

Preferences about physical activity were sometimes the first indicator of a child's gender identity. One parent recounts the day that their foster child, then presumed to be a boy, came to live with them:

she took all her soccer gear, her shoes, the clothes, everything, and walked up and put them in our rubbish bin and said, “I'm never playing soccer again”. Oh, that's quite a statement from someone who's been through such a traumatic day. You know? What, what was that about? And then, you know, getting to talk with [our child]. She told us how it was her dream to do dance, all she ever wanted to do was go to dance. Could we take her to dance? (Tynan and Melissa, child, she).

This child did not yet have the language to explain to new foster parents their aspirations for gender expression, and how their gender identity had been over-ruled in the past. Despite this, they were able to act with agency, and throw away their soccer gear. This was such a brave thing to do, for a child that had already been rejected from a previous foster home, to give her new carers such a strong declaration of her desired gender expression in the way she had available to her. This action opened up new possibilities for her relationship with their foster parents, who then asked themselves—“what was that about?”. This led to participation in dance, and subsequently gender affirmation and transition. These parents provided several anecdotes of their child's gender euphoria when dancing in public spaces, describing her as “having the time of her life” (Tynan and Melissa, child, she).

Children's choices about what to wear for dance and sport provided further information about gender identity. In the early years, the participation of presumed cis boys in dance classes was supported through them wearing shorts rather than tutus. Trans girls often began participating in dance wearing shorts:

I look back and she was the only one in a different outfit on stage. (Renata, preschooler, she)

In hindsight, this parent understands that gendered expectations about what to wear restricted her child's participation in dance:

[She] did a couple of [ballet] lessons. And she didn't want to go back. Because she wouldn't wear the other [boys'] uniform. … At that point, I didn't know what was happening. (Paige and Tom, preschooler, she)

Dance schools were often supportive of presumed cis boys wearing “the same thing as the girls” (Tynan and Melissa, child, she). As a child's gender diversity became clearer, dance schools might provide a choice about costume. For example, one parent explained “when there was a boy or a girl costume, she would be asked ‘which one would you like to wear?’” (Aurora and James, child, she/they). Over time, children and parents test out what agency they have to change the activities, and what to wear.

Children want to play sports with their friends, and having same gender friends can be an important component of gender affirmation. One young person left his sports team as part of his social transition. His parent explained:

he did netball … the minute he changes [socially transitioned] he's like “I'm not going to be the only guy on the court”. (Sylvie, youth, he)

Another child:

certainly didn't connect with the boys despite our attempts, and we would do things like put [her/them] in soccer and organize play dates and cricket and all the things that boys were doing (Aurora and James, child, she/they)

At the same time,

[she/they] was also part of a little dance group from the time [she/they] was two or something, and would always wear [her/their] tutu to dance and was part of that crew till [she/they] was 11 (Aurora and James, child, she/they)

Children's preferences for participation that communicated and affirmed gender identity also encompassed seeking community.

In sports environments dominated by binary cisgenderism many children sought out sports that are traditionally associated with their preferred gender identity. Children who were clear about their binary gender identity sought affirmation of this through their choice of participation in physical activity. Parents who were interviewed did not themselves subscribe to sports participation being restricted by gender, in that girls should participate in feminised activities like dance and boys should participate in masculinised activities like football (32). Instead, the parents sought to understand what their child wanted and to create opportunities accordingly for their child to make choices about participation from a range of possibilities.

Not all children could communicate or affirm their gender through a choice of binary sport. The parent of a non-binary young person explained:

Everything's binary, … the girls' team or the boy team. Which one are you on? … So even just that was so difficult to navigate, (Bree, youth, they)

This binary structuration of sport made it difficult for non-binary children to participate. Instead, some exercise agency through normative resistance (28) and “refuse to play” (Raine, child, they) rather than make a binary choice. While binary trans people may seek to pass to have their gender affirmed (24), non-binary children cannot affirm their gender within a binary structuration.

The availability of mixed gender sports teams was an enabler of participation for some. This was sometimes because they were non-binary, sometimes because they were still navigating how and in what circumstances they wanted to disclose their transitude. For example, one parent explained that their young person, presenting as the boy they were assigned at birth in late high school, “didn't want to do all the usual sports like cricket or soccer [or rugby]” (Ayla, youth, she). Instead, she tried jujitsu and taekwondo, finally finding volleyball, which she enjoyed, and was good at. This young woman was navigating her transition, living with the gender identity presentation of a boy during the last years of high school, deferring transition until she left school. Notably, the sports she chose were less strongly gendered, with mixed teams or individual participation. This inventive pragmatism (28) enabled her to participate in such a way that binary gender was not amplified by the sport or team in which she played.

In conclusion, children communicate their gender identity in their navigation of sports and dance choices, what to wear, and the communities where they feel comfortable. This paper is not arguing that all children who resist gender norms are trans, but that gender norms are the structure within which many trans children agentically communicate their gender identity. Children and their parents learn about the gendered structure of sports participation and exercise agency through choices that affirm children's gender identities through the subjectivities that are available to them (27), deploying both normative resistance and inventive pragmatism (28).

### 3.2 Navigating disclosure and privacy

A recurring theme in parent accounts was under what conditions should they or their child disclose or conceal the child's transitude in order to facilitate participation in physical activity. Navigating the administration of enrolment was one example of this. Others were determining appropriate clothing, and finding a balance in community.

Parents described an administrative burden of problem solving to navigate enrolment processes and sporting body policies. In some instances, parents reported that online enrolment systems asked for sex assigned at birth. One parent recalled being asked to bring their child's birth certificate and stand in line with other parents to enrol, to provide evidence of sex assigned at birth. This parent did not want to disclose their child's sex assigned at birth, particularly in a public place. Instead, in the absence of gender affirming documentation, they went to the police station to sign an affidavit that they had lost the birth certificate, and that their daughter was a girl. To the puzzled police officer, the parent explained “my daughter is trans, and this is how I can get her enrolled in [sport]” (Janice, child, she). This disclosure, an example of inventive pragmatism (28), was preferable to a public disclosure of transitude in a community setting.

Navigating what children wore to participate in sport and dance was commonly discussed by parents, not only as a way to communicate and affirm gender, but also with respect to disclosure. Activities where clothing might reveal bodily sex characteristics that are not normatively associated with a child's gender identity required parents to take special care. This was particularly the case for swimming, gymnastics and dance, “mainly because a leotard is required or [swimming] costume” (Paige and Tom, preschooler, she). Care was required because children did not want to be hypervisible as trans. Having a private place where children could change was important for some parents:

[In this activity] she would have to get wet and in wetsuits and stuff and get out so it was changing involved and I said … “can you make sure she can have a private place to change” that's really all I cared about and they were really supportive. (Bea, child, she)

Whilst discussion about clothing was congruent with clothing having been identified as a barrier to participation by other research, there was less discussion of changing rooms than one might expect from the literature (17). This may be because many of these children were young, and they are already in correct attire when they arrive at the sports venue. Notably, Bea has highlighted a time when a parent is not present. Where they have a parent with them, in community settings, changing facilities are less problematic.

Where children socially transitioned within a community, the possibility of not disclosing transitude was not available. The possibility for not disclosing became available for binary trans children if the family moved, or joined a community after a social transition. Non-disclosure was the choice made by Bea's child:

No one knows. So, where we used to live she was just out and seemed ok with that but that was when we lived [elsewhere] and then we moved to [here]. (Bea, child, she)

Reflecting on the impact of this decision by the child to be private, the parent continues:

I actually think she was happier when people knew. Her anxiety gets a lot worse when I think she feels like there's a secret or she's hiding something. Or that people won't truly accept her. (Bea, child, she)

Navigating the administrative requirements to enable participation in a way that was congruent with children's gender identity, and protecting privacy, was challenging for parents. In order to facilitate participation, parents had to problem solve, to understand what their child wanted, and work out how to make this happen. The data supports disclosure or privacy not being a once off decision (24).

Some parents felt that making sure the community of parents knew might pre-empt problems later, others felt non-disclosure was more appropriate. Parents sometimes felt pressure from other parents in the community to be more public in their declaration of a child's transitude, to “make an announcement” (Tynan and Melissa, child, she). Melissa pushed back on this pressure, comparing their child's transitude with also living with a disability. She recounted this exchange with another mother whilst having coffee with a group of parents:

[she said to me] “why couldn't you have sent everyone's parents a letter?” I said “a letter? What about?” “About what you're doing!” I said, I literally said this: “Did you send a letter to everyone that your daughter had Down's syndrome? … because I didn't send a letter to everyone that [our child] had cerebral palsy.” (Melissa, child, she)

The topic is now unspoken between them: “I just don't bring it up. And she doesn't.” (Melissa, child, she). This reminds us that families might live in the same community with people who are not supportive for years. This middle ground, where some people knew and some didn't, allowed the child to feel supported to participate. Similarly, for another family, a comfortable balance was where “management don't give a hoot, and most people don't know” (Paige and Tom, child, she). Because trans kids playing sport is contested, parents and children face a choice about whether and how to be stealth, and at the other extreme, to announce the participation of their trans child to the other families in the community.

Van der Wal's (24) argument that trans people are in an iterative process of disclosure and concealment is applicable here in childhood, where children cannot avoid cis-normative spaces like schools, and have to declare gender to participate in sport, and navigate accompanying administrative processes. Furthermore, trans children are likely to transition their gender within a community that previously knew them by their name and gender assigned at birth. In response, parents hold a space that balances the ability for privacy with exploration and navigation of what is possible. Exploration can imply that children do not know their gender identity, or that it is fluid, which is not my argument here. This space for exploration is in regard to navigating the identities that are possible, and the policies and communities that enable participation. Parents seek a place where their child's transitude is unremarkable, the sport or dance community and administration may know, and their child is supported to participate.

### 3.3 The kindness of community

Social participation in community was an important element of sport and dance activity. One parent described how her child had been to a camp organised by an organisation that supports LGBTQIA+ young people. At this camp:

[There were] teens, and a lot of LGBTQ camp councillors. They do craft, they play sport. It's brilliant. They had the best time. And he still sort of comes across people that he knew from that and you know he really made friends from it. He came home just buzzing. (Margaret, youth, he)

Not only did this teen have a great time at camp and made friends, shortly afterwards he was able to tell his parents that he was trans. Another parent explained the euphoria for her teen in attending a trans and gender diverse pool party organised by a local council.

It was a big deal for him to go and, and he said: “No, I don't want to take any friends.” But he wanted to invite his guncles. It's my best mate. Gay dads and their kids. So yeah, they came and we had such a fantastic day. … it was fantastic for [kiddo] to see these normal family units, you know, with gay dads, with the top surgery with their shirt, like they're shirtless, you know, wearing their board shorts, and, you know, just having a beautiful day like everyone was. And so I just loved seeing him so happy. And all these people, and older people, that were just like him. And younger people having so much [fun]. (Kim, youth, he)

Swimming is an integral part of Australian culture and childhood. However, this teen had reached the point where he would only swim when he was alone, at home. The pool party was an opportunity to enjoy swimming once more in community. These stories are congruent with others' findings that queer contexts can enable trans people to participate in sports (33).

In most Australia primary schools, the swimming carnival is an important annual event. Regarding their child's participating in the school swimming carnival, one parent recognised that she herself was:

“fearful around swimming sports, wondering where [child name] was gonna have to get changed like, is there gonna be a place for [child name] to get changed? Is there, are people going to tease [child name] about the bathers that they're wearing?” (Raine, child, they)

Because of these fears, the community response from other parents and children was important. Parents valued responses based on kindness and respect, and were impacted by comments and questions that they perceived to be hostile or being an unsafe environment for their child.

I do remember the day that she first wore the tutu to dance. And I just remember a couple of the mums like, I can’t remember exactly what they said. And it's always the parents more than the kids. I can't remember what this mum said, I actually got upset. I left in tears. (Renata, preschooler, she)

It is the accumulation of experiences like this that build the emotional load for parents.

at that point I had I'd received a fair bit of negative comments and everything else, I was feeling really nervous … not just at swimming but in general (Paige and Tom, preschooler, she)

There is fear involved because of negative reactions from others. Another parent, also fearful, recognised that this may be their fear rather than their child's:

And still, with gymnastics, It's a fear for me, it's not from her, with uniforms. (Paige and Tom, child, she)

Sometimes, in a community setting, being open about a child's transitude allowed others to seek advice. One parent recounted how she was subsequently approached by another parent, seeking advice on behalf of another trans child. This adds to the emotional work that parents do.

Parents being physically present also helped parents manage social participation. Tynan and Melissa were apprehensive about the dress her child wanted to wear to dance at the school disco, and tried to dissuade her:

She stood her ground, [saying] “I don't care”. And I said, “what if someone comes up to you? And says, Why are you in a dress? What are you going to do?” “I'm just going to ignore them.’ I said, “What if nobody wants to dance with you? What if nobody wants to talk with you? Because you're in a dress?” She said – “you'll be there”. (Tynan and Melissa, child, she)

Another parent was planning how to make the experience of the school swimming carnival positive for their child. They were “fearful” about bullying, changing room provision, and the child's swimwear. They were heartened that the school said “You can come along if you want to help them” (Raine, child, they). These examples highlight the importance to children of parental presence in navigating this landscape.

### 3.4 “Give it a red hot crack”

Competition was not something that parents talked about much, despite competition dominating mainstream discourses about trans participation in sport (9). Instead, parents are seeking community and participation for their children. Often, at the school swimming carnival, the whole school is expected to attend, and children compete in races to win points for their house. One parent contrasted their previous school's approach to the swimming carnival with their current school's approach:

At the old primary school at the swimming carnival we used to get this ridiculous memo that said unless your kid's in [the competitive swimming] squad and they are a competitive swimmer, don't send them. “This is not a splash and play activity”. I don't need to explain how wrong it is. Here [at the new school] they basically said there will be a race for everyone. Everyone will have the opportunity to score a point for their house. Come and give it a red hot crack. And I think that typifies the whole. The people who are already in squad don't need special events. (Janice, child, she)

Another parent gave this much subtler account of the value placed on competitiveness:

Paige: I would always strategically pack costumes with longer frills … we got away with it for a long time. Until one day, the swim instructor came up and was like, a super supportive gentleman. He was like, “Look, I've seen your story”. (I was in paper at one point.) “But I feel like the longer frills or the long rashies are holding her back.” Oh no!

Interviewer: in her physical swimming ability?

Paige: physical ability. And I said, Well, you know why she wears those?' And he was like, “Yeah, I know, have a think about it”. (child, she)

This example of competitive discourse prioritises sports skill development over participation and comfort.

At the same time, this child did aspire to elite competition in another field. This was very much a concern for her parents, who were planning how this could be possible.

we have to get on to [accessing medical transition] as soon as possible because she's doing competitive [sport]. And with everything I've read, you know, they have to be on blockers before they start puberty in order to compete at an elite level. (Paige and Tom, child, she)

For those that do excel at sport, they are conscious of a need to navigate discourses of competition and gender affirming healthcare that opens up a pathway to be able to keep playing at an elite level. Parents of trans children know that the onset of puberty is a major milestone for which they have to prepare (15). In this instance, the spectre of competitive sports participation amplifies parent concerns about accessing medical affirmation as early as possible.

Children and young people do reach a point where they no longer “give it a red hot crack”. Sylvie had two explanations for why her child stopped participating in sports. The first was that “he was probably getting to an age where he was phasing out of that stuff anyway” (Sylvie, youth, he). Then, she elaborated, saying “subsequently, the [poor] mental health led to a lot of fatigue and stuff that just made it all of that stuff hard anyway, - just too tired” (Sylvie, youth, he). We saw above that children might opt out of physical activity when the gender stereotypes, uniform or community are not experienced as affirming. Children also opt out of sport when the mental health impacts of other people's responses make them tired.

We know that there is divisive debate about trans people in elite sports, particularly trans women competing in women's sport. The battle lines are drawn over what is fair in competition, particularly, whether it is fair that trans women, presumed to have an advantage in body size and strength, should compete against ciswomen (9). It is not the place of this paper to catalogue and address these discourses, but to note that these discourses are a wider context when parents navigate community sport with their children. Whilst parents did not discuss having to counter this argument in children's sports, there were indicators that the discourses of competition start young.

## 4 Conclusion

Parents work hard to enable participation for their trans children. This work encompasses finding supportive community, navigating administrative requirements and solving the problem of what to wear. Parents combine normative resistance and inventive pragmatism (28). Children's agency in sport and dance participation is enacted through the choice of activity, clothing and community, and operates to communicate and affirm their gender identity. There are more opportunities for binary trans children to do this than for non-binary children, due to the cis-normative organisation of sport and dance. This structural cisnormativity is the background within which agency is shaped and constrained.

Participating in gendered sports is an important way for binary trans children to be affirmed in their gender. Opportunities for mixed gendered sporting environments are also crucial. They reduce the need to conform to gendered expectations, create a space of more ambiguous gender identity, and support inclusion of non-binary children. Access to sport is most important to parents for participation and community, rather than competition.

This research indicates that participation of trans children and young people could be increased through opportunities that do not amplify binary gender structures, such as more mixed gender teams, and a reduction in sorting by gender, particularly prior to puberty. Flexibility in the choice of what to wear to participate, and prioritising participation over competitiveness would be beneficial. Clear policy statements about inclusion in policy and communities would set the tone for community responses. Participation could also be improved through removing any requirement for gendered identity documents for enrolment. Sports and physical activity in queer space also support participation.

A limitation of this work is that it relies only on parent accounts. The perspectives of trans young people themselves, administrators and coaches in children's community sport and dance, and other parents in these communities would add to our knowledge of how to navigate and change cis-normative binary environments and mitigate the wider culture of division over trans participation in physical activity.

## Data Availability

The datasets presented in this article are not readily available because under the ethics approval conditions, data can only be used by the same researcher and will be destroyed after five years. Requests to access the datasets should be directed to c.townley@westernsydney.edu.au.
